# Influence of support on intra-abdominal pressure, hepatic kinetics of indocyanine green and extravascular lung water during prone positioning in patients with ARDS: a randomized crossover study

**DOI:** 10.1186/cc3513

**Published:** 2005-03-31

**Authors:** Pierre Michelet, Antoine Roch, Marc Gainnier, Jean-Marie Sainty, Jean-Pierre Auffray, Laurent Papazian

**Affiliations:** 1 Service de Réanimation Chirurgicale, Hôpitaux Sud, Marseille, France

## Abstract

**Introduction:**

Prone positioning (PP) on an air-cushioned mattress is associated with a limited increase in intra-abdominal pressure (IAP) and an absence of organ dysfunction. The respective influence of posture by itself and the type of mattress on these limited modifications during the PP procedure remains unclear. The aim of this study was to evaluate whether the type of support modifies IAP, extravascular lung water (EVLW) and the plasma disappearance rate of indocyanine green (PDR_ICG_) during PP.

**Methods:**

A prospective, randomized, crossover study of 20 patients with acute respiratory distress syndrome (ARDS) was conducted in a medical intensive care unit in a teaching hospital. Measurements were made at baseline and repeated after 1 and 6 hours of two randomized periods of 6 hours of PP with one of two support types: conventional foam mattress or air-cushioned mattress.

**Results:**

After logarithmic transformation of the data, an analysis of variance (ANOVA) showed that IAP and PDR_ICG _were significantly influenced by the type of support during PP with an increase in IAP (*P *< 0.05 by ANOVA) and a decrease in PDR_ICG _on the foam mattress (*P *< 0.05 by ANOVA). Conversely, the measurements of EVLW did not show significant modification between the two supports whatever the posture. The ratio of the arterial oxygen tension to the fraction of inspired oxygen significantly increased in PP (*P *< 0.0001 by ANOVA) without any influence of the support.

**Conclusion:**

In comparison with a conventional foam mattress, the use of an air-cushioned mattress limited the increase in IAP and prevented the decrease in PDR_ICG _related to PP in patients with ARDS. Conversely, the type of support did not influence EVLW or oxygenation.

## Introduction

Prone positioning (PP) improves arterial oxygenation in 50 to 75% of patients presenting with acute respiratory distress syndrome (ARDS) [1]. Although this postural treatment is currently considered simple and safe [1], the restriction of abdominal movements during PP is associated with an increase in intra-abdominal pressure (IAP) [2-5] with potential adverse effects on hemodynamic status and splanchnic perfusion [6-8]. Several studies have evaluated the clinical implications of this side effect of PP [4,5,9]. They reported significant but limited increases in IAP without impairment of cardiopulmonary, renal or hepatosplanchnic functions during short periods of PP. However, all patients included in these studies were placed on air-cushioned beds, which might have reduced the restriction of abdominal movement during PP [4,5,9] compared with a conventional foam mattress. Indeed, the air-cushioned mattress reduced interface pressure to a greater extent than the foam mattress [10,11]. Moreover, the addition of pillows under the thorax and the pelvis did not produce a decrease in IAP during the PP procedure with a foam mattress [12]. Because no direct comparison has been made between different supports, the respective influences of the type of support and the posture itself on IAP and its related potential adverse effects remain unclear. Therefore, in the perspective of standardization, the question of the interest of a special device before the institution of PP should be clarified [1].

The aim of the present study was to investigate whether the evolution of IAP, liver function assessed by the plasma disappearance rate of indocyanine green (PDR_ICG_) [5] and extra-vascular lung water is related to the type of support during PP. We therefore prospectively compared, in a population of medical-ARDS patients, the effects of an air-cushioned mattress and a conventional foam mattress during PP.

## Methods

### Patients

Twenty consecutive patients with ARDS were included and turned prone in the medical intensive care unit of Sainte Marguerite University Hospital in Marseille, France. Patients were prospectively included in this study after obtaining written informed consent from the next of kin. The study design was approved by the Comité Consultatif de Protection des Personnes dans la Recherche Biomédicale of Marseille. ARDS was defined in accordance with the recommendations of the American–European Consensus Conference [13]. Patients with unstable cardiovascular function, cerebral injury or unstable spinal fractures, patients subjected to major abdominal surgery and patients with a history of neuromuscular disease were excluded.

### Sedation, catheters and ventilation

All patients were sedated and paralyzed by the continuous infusion of sufentanil, midazolam and cisatracurium throughout the study period and were ventilated with conventional volume-controlled mechanical ventilation (7200 series or 840, Mallinckrodt Puritan Bennett, Carlsbad, CA, USA).

Respiratory and hemodynamic status was stable for 12 hours before inclusion. When patient received vasoactive drugs, the rate of infusion was kept stable throughout the study. On inclusion into the study, the mean tidal volume was 6.9 ± 1.9 ml/kg, the mean respiratory rate was 20 ± 4 cycles/min (the respiratory rate was adjusted to maintain a constant minute ventilation throughout the study period) and the positive end-expiratory pressure was 11.3 ± 2.0 cmH_2_O. The selection of the appropriate PEEP level was performed by increasing PEEP in steps of 2 cmH_2_O. A blood gas analysis was performed after a 30 min period of stabilization of blood oxygen saturation. Finally, the lower level of PEEP giving the greater improvement of oxygenation was chosen. The levels of PEEP, the tidal volume and a fraction of inspired oxygen (FiO_2_) of 0.8 were maintained constant throughout the study period.

A pulmonary artery catheter (Baxter Healthcare Corporation, Irvine, CA, USA) was placed in all patients. It was inserted percutaneously through the right jugular or left subclavian vein and positioned with the distal port in the pulmonary artery and the proximal port in the right atrium. For measurements of the PDR_ICG _and extravascular lung water (EVLW), a 5F catheter was placed into the left femoral artery, together with a thermistor-tipped fiber-optic catheter (Pulsiocath, 4F, FT, PV-2024-L; Pulsion Medical System, Munich, Germany) which was advanced into the descending aorta.

### Support surfaces

The control surface was a three-piece molded foam mattress (APLOT^®^; Asklé, Nîmes, France). The specialist air mattress was a dynamic alternating cells design, with automatic adjustment for patient weight (ProNimbus^®^; Huntleigh Healthcare, Luton, UK).

### Measurements

#### Hemodynamic parameters

Routine invasive hemodynamic monitoring included arterial and pulmonary artery thermodilution catheters. Systemic and pulmonary arterial pressures, pulmonary artery occlusion pressure and right atrial pressure were measured at end-expiration. The midaxillary line was taken as the zero reference point in the supine and prone positions. Cardiac index, venous admixture and pulmonary vascular resistance were calculated from conventional formulas.

#### Blood gas analysis

Systemic and pulmonary arterial blood samples were withdrawn simultaneously within 3 min after the measurement of cardiac output. Arterial pH, arterial oxygen tension (PaO_2_) and arterial CO_2 _tension were measured with a blood gas analyzer (278-blood gas system; Ciba Corning, Medfield, MA, USA). Arterial and mixed venous oxygen saturation (SaO_2 _and SvO_2_, respectively) were measured with a calibrated hemoximeter (270-CO-oxymeter; Ciba Corning).

#### Respiratory parameters

The respiratory parameters measured were compliance of the respiratory system, exhaled tidal volume, peak inspiratory pressure, mean inspiratory pressure, and respiratory rate. Quasi-static compliance of the total respiratory system was obtained by dividing the tidal volume by the difference between plateau pressure and the total PEEP according to the method described by Gattinoni and colleagues [14].

#### Thoracic volumes, EVLW and hepatosplanchnic perfusion measurements

The transpulmonary indicator dilution technique was used to determine the intrathoracic blood volume, the EVLW and the PDR_ICG _[15]. PDR_ICG _is derived from the half-life of indocyanine green and reflects the percentage of the initial plasma dye level eliminated by the liver [16]. A thermistor-tipped fiber-optic catheter placed in the descending aorta detected the dye and temperature dilution curves. The EVLW and the PDR_ICG _were automatically calculated by a computer (Pulsion Cold Z-021; Pulsion Medical System) from the average of three measurements [16].

#### Measurement of IAP

IAP was measured with a transurethral bladder catheter [17]. Normal saline (100 ml) was infused through the urinary catheter into the bladder. The catheter was then clamped and the IAP was recorded by a pressure transducer as mean pressure at end-expiration. Zero was set at the level of the pubis in both positions.

### Protocol

Baseline measurements were performed in the supine position after 1 hour of steady-state conventional mechanical ventilation. Then the following two periods of PP were randomized: 6 hours of PP on the moulded foam mattress, and 6 hours of PP on the air-cushioned mattress. A period of 18 h in the supine position separated the two periods in the prone position. Each patient was his or her own control. Measurements were achieved in the supine position, after 1 and 6 hours of PP.

Change in position was performed manually by three nurses and two staff members. In the prone position, the arms were laid parallel to the body. Care was taken to avoid eye damage and any non-physiological movements of the limbs during posture changes. Whatever type of support was used, no pillow was used to support regions such as the chest or pelvis.

Measurements were performed before, after 1 hour and after 6 hours of each period of PP.

### Statistical analysis

Statistical calculation was performed with the Sigma Stat 3.0 package (SPSS Inc., Chicago, IL, USA). Distribution was checked. Data were expressed as mean ± SD if the distribution was normal and as medians and interquartile range if the distribution was not normal. Significant differences were analyzed by general factorial analysis of variance (ANOVA) with a prior logarithmic transformation when required (non-parametric distribution). For intra-group changes, Tukey's test for multiple comparisons was applied to compare the variations between supports and different times of the study. For serum transaminases and creatinine, a Mann–Whitney *U*-test was used to compare the values before and after the protocol. When a correlation was calculated, Pearson's coefficient of correlation was used. When distribution was not normal, Spearman's rank correlation was used.

*P *< 0.05 was considered significant.

## Results

Characteristics of the population are listed in Table 1. On admission, the mean Simplified Acute Physiologic Score II (SAPS II) score was 52 ± 12 and the severity of ARDS was assessed by a lung injury score of more than 2.5 in all patients (3.1 ± 0.3). The mortality rate for the 20 patients (15 men, 5 women; age 53 ± 12) was 25%. Among the patients enrolled in the study, six patients received norepinephrine (noradrenaline) (dose 0.3 ± 0.2 μg/kg per min) and one patient received epinephrine (adrenaline) (0.4 μg/kg per min) on inclusion. No modification of the infusion rate of norepinephrine and epinephrine and no fluid expansion were undertaken during the study period.

### Effects of prone position and support on IAP, hepatosplanchnic function and EVLW

**Figure 1:**
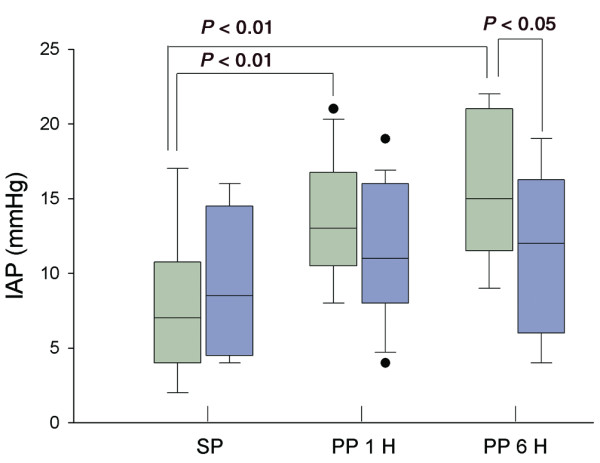
Effects of both supports on intra-abdominal pressure (IAP). The different durations of study are reported along the *X*-axis: SP, PP 1 H and PP 6 H represent baseline in supine position, prone position at 1 hour and prone position at 6 hours, respectively. Pale gray boxes, foam mattress; dark gray boxes, air-cushion mattress. Each box plot represents the median, the 25th and 75th centiles, and the largest and smallest values that are not outliers. Outliers are represented as filled circles. *P *< 0.01 and *P *< 0.05 by Tukey's post-hoc test.

**Figure 2:**
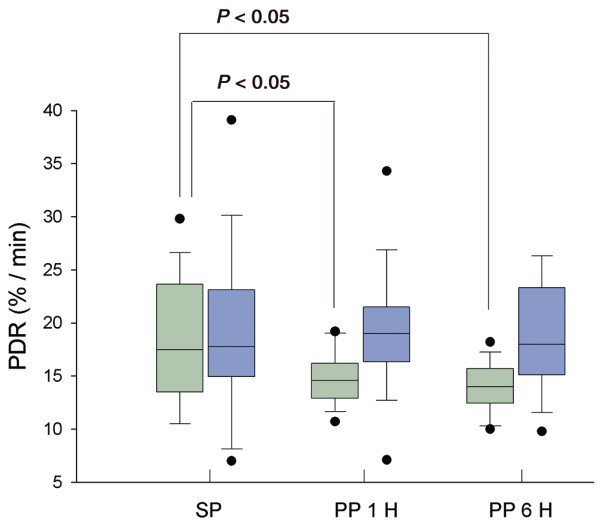
Effects of both supports on the plasma disappearance rate (PDR) of indocyanine green. The different durations of study are reported along the *x*-axis: SP, PP 1 H and PP 6 H represent baseline in supine position, prone position at 1 hour and prone position at 6 hours, respectively. Pale gray box, foam mattress; dark gray box, air-cushion mattress. Each box plot represents the median, the 25th and 75th centiles, and the largest and smallest values that are not outliers. Outliers are represented as filled circles. *P *< 0.05 by Tukey's post-hoc test

A logarithmic transformation of the data led to normally distributed values of IAP and PDR_ICG_. We therefore performed a two-way ANOVA that showed an increase in IAP after 1 and 6 hours of PP on a foam mattress in comparison with baseline values (*P *< 0.01 by Tukey's test; Fig. 1) whereas it remained unchanged when patients were positioned on a specialist air mattress. IAP was higher on a foam mattress after 6 hours of PP in comparison with a specialist air mattress (*P *< 0.05 by Tukey's test; Fig. 1). PDR_ICG _decreased after 1 and 6 hours of PP on a foam mattress in comparison with baseline values (*P *< 0.05 by Tukey's test, Table 2 and Fig. 2) whereas it remained unchanged when patients were positioned on a specialist air mattress.

There was no correlation between changes in IAP and PDR_ICG_. Furthermore, the analysis of hepatic and renal variables did not show any statistical variation after the PP procedure period (Table 3).

There was no modification in EVLW and intrathoracic blood volume related to posture or support changes (Table 2).

### Effects of prone position and support on gas exchange (Table 2)

PP induced an increase in the PaO_2_/FiO_2 _ratio (*P *< 0.001 by ANOVA) regardless of the type of support. There was no correlation between evolution in PaO_2 _and changes in IAP. PP reduced the true pulmonary shunt (*P *< 0.05 by ANOVA) without any influence of the kind of support.

### Effects of prone position and support on hemodynamic and respiratory parameters (Table 2)

ANOVA showed that CVP, mean pulmonary arterial pressure and pulmonary artery occlusion pressure increased significantly in PP (*P *< 0.001) without any influence of the kind of support. Although PP induced a significant reduction in the static compliance of the total respiratory system (*P *= 0.007 by ANOVA), these modifications were not influenced by the kind of support. Other hemodynamic or respiratory parameters were not affected by prone position or type of support.

## Discussion

The results of this study indicate that the use of an air-cushioned mattress for the PP procedure limited the increase of IAP and prevented a decrease in PDR_ICG _related to PP. Nevertheless, these modifications were not associated with differences between supports for EVLW, oxygenation or cardiovascular parameters.

Even if the routine use of PP did not improve the survival of patients with ARDS in a recent multicenter randomized trial [18], it could be considered useful in the more hypoxemic patients. The improvement in oxygenation produced by PP is often related to an increase in aerated lung tissue with a decrease in venous admixture and a decrease in thoraco-abdominal compliance [19]. On the basis of these considerations, one can presume that the more the thoraco-abdominal wall is restricted during PP, the greater the gain in arterial oxygenation that should be obtained [4]. Nevertheless, in different settings, turning patients prone has been reported to induce an increase in IAP with potential side effects on cardiopulmonary, renal and hepatosplanchnic function [7,8]. The impaired hepatosplanchnic perfusion could lead to the development of multiple system organ failure [7,20,21], increasing the mortality rate of patients with ARDS [22,23].

Several studies were therefore designed to investigate the effects of PP on IAP modifications and clinical consequences [4,5,9]. The cumulative results of these studies indicate that, despite a small increase in IAP, PP improves arterial oxygenation without affecting cardiopulmonary, renal or hepatosplanchnic function. Nevertheless, the constant use of air-cushioned beds during PP in these studies could have contributed to limiting the effects of PP on IAP [5]. Indeed, this specialist air mattress presents a dynamic alternating-cells design with automatic adjustment for patient weight and a redistribution in pressure from the heavy parts (abdomen) to other areas. This support has been extensively reported as providing the lowest interface pressure in comparison with other supports, including foam mattresses [10,11]. Conversely, the use of a foam rubber mattress has recently been associated with an increase in IAP during PP without further reduction when pillows were added under the thorax and the pelvis [12].

Similarly, in our study, the use of foam rubber mattresses during PP induced a significant increase in IAP with a concomitant reduction in PDR_ICG_, whereas air-cushion mattresses did not do so. Although these findings seem to indicate a superiority of the air-cushion support in terms of abdomen release, the clinical consequences of such modifications should be interpreted with regard to their duration. Indeed, despite an increase in IAP reaching 15 mmHg after 6 hours of PP on a foam mattress, the differences in liver function evaluated by PDR_ICG _were limited during the PP procedure and did not induce significant modifications in hepatic biological variables during the period after PP. Conversely, although previous results have shown that a higher level must be reached to induce clinical modifications [24,25], a recent multicenter epidemiological study has reported the detrimental influence of a prolonged increase in IAP even for such moderate levels as 12 mmHg [26].

Another consequence of an increase in IAP during PP could be the modification of EVLW. Indeed, a decrease in EVLW has been proposed as an alternative mechanism that might account for the benefit related to PP [27]. Furthermore, an increase in IAP has recently been reported to worsen pulmonary edema in a lung injury induced by oleic acid [28]. Despite a PP-related increase in IAP with the foam mattress, our results did not demonstrate a significant modification in EVLW. The basal levels and the limited differences in IAP between the two supports probably explained the lack of influence of support on pulmonary edema assessed by EVLW in our study. An alternative, although not mutually exclusive, explanation for the observed evolution in EVLW could result from the underestimation of edema by this technique, because several areas of the lung are underperfused during ARDS [29].

Because the first objective of PP is still the improvement of ARDS-related hypoxemia, the potential influence of the support on oxygenation evolution represents another subject of concern [19,30,31]. Our results confirm previous data on PP-related oxygenation improvement but do not report any difference between the supports. The fact that the type of support does not appreciably influence the effects of PP on gas exchange has been deduced from previously published studies. Although Pelosi and colleagues reported an improvement in PaO_2 _that was correlated with a decrease in thoraco-abdominal compliance [19], these results were interpreted as being principally related to the modification of the chest-wall component of the thoraco-abdominal compliance during PP, without significant intervention of the abdominal part. Furthermore, Colmenero-Ruiz and colleagues, in an experimental study, showed that abdomen release in PP does not induce significant modification in oxygenation [31].

However, it should be noted that all patients included in the present study presented a medical etiology for their ARDS with an IAP in the supine position that was expected to be lower than in a surgical patient population. Consequently, our results must be interpreted with regard to our selected population because the effects of different support during PP could be different in patients with increased IAP (especially in surgical patients). Indeed, Gattinoni and colleagues [32] reported an IAP ranging from 6 mmHg in patients with ARDS of pulmonary cause to 16 mmHg in patients presenting with ARDS of extrapulmonary cause. The basal level of IAP and the magnitude of changes during PP for surgical patients could induce more relevant results than in our study and imply the need for further investigation in this population.

Our results suggest that the limited modifications in cardiovascular, renal or hepatosplanchnic function observed during PP are probably not related to the type of support but most probably to the relative harmlessness of this postural technique because patients do not present abdominal hypertension before prone positioning.

## Conclusion

Consequently, the use of an air-cushion mattress for PP seems to be unnecessary in a standardized protocol in medical patients. However, the use of an air-cushion mattress is still of particular interest in reducing the incidence of pressure ulcers when prolonged periods of PP are needed and in facilitating the PP procedure for tracheostomized patients. Nevertheless, because the duration of PP period was limited to 6 hours in our protocol, a specific comparison between the two supports regarding skin lesions was not made in the present study and will require further evaluation.

## Key messages

• 	In a population of medical-ARDS patients, the evolution of IAP and liver function during prone positioning is partly related to the type of support.

• 	The use of an air-cushion mattress do not influence the oxygenation and EVLW evolution during prone positioning.

## Abbreviations

ANOVA = analysis of variance; ARDS = acute respiratory distress syndrome; EVLW = extravascular lung water; FiO_2 _= fraction of inspired oxygen; IAP = intra-abdominal pressure; PaO_2 _= arterial oxygen tension; PDR_ICG _= plasma disappearance rate of indocyanine green; PEEP = positive end-expiratory pressure; PP = prone positioning.

## Competing interests

The author(s) declare that they have no competing interests.

## Authors' contributions

PM, AR and LP were the principal investigators and led the conceptual design of the design and the manuscript preparation. MG made contributions to the acquisition of data and to the analysis and interpretation of data. JMS, JPA and LP performed a first manuscript revision and gave final approval of the version to be submitted. All authors read and approved the final manuscript.

## Figures and Tables

**Table 1:** Characteristics of the population

Patient no.	Age (years)	Sex	BMI	LIS	SAPS II	ARDS duration (days)	Causes of ARDS	Outcome
1	51	M	42	3.0	39	4	Bacterial pneumonia	Alive
2	39	M	27	3.0	60	1	Aspiration pneumonia	Alive
3	65	M	33	3.3	46	1	Cytomegalovirus pneumonia	Alive
4	64	M	28	2.7	71	2	Bacterial pneumonia	Alive
5	50	F	23	2.7	49	7	Septic shock	Died
6	44	M	24	3.3	45	1	Bacterial pneumonia	Alive
7	71	M	26	3.6	51	7	Bacterial pneumonia	Died
8	37	M	34	3.2	30	3	Lung contusion	Alive
9	45	M	25	3.3	62	1	Bacterial pneumonia	Died
10	37	M	25	3.2	74	6	Community-acquired pneumonia	Alive
11	52	F	37	2.7	52	4	Aspiration pneumonia	Alive
12	49	M	25	2.7	40	2	Bacterial pneumonia	Alive
13	70	M	28	3.2	57	4	Bacterial pneumonia	Alive
14	48	F	31	3.5	72	1	Aspiration pneumonia	Alive
15	60	F	27	3.2	58	2	Septic shock	Alive
16	48	M	29	3.7	44	7	Bacterial pneumonia	Alive
17	73	M	38	3.3	62	4	Aspiration pneumonia	Died
18	56	M	25	2.7	56	3	Community-acquired pneumonia	Alive
19	34	F	28	2.5	32	3	Bacterial pneumonia	Alive
20	65	M	23	3.3	48	2	Aspiration pneumonia	Died

ARDS duration, duration of acute respiratory distress syndrome before inclusion BMI, body mass index; LIS, lung severity score; SAPS II, Simplified Acute Physiologic Score II

**Table 2:** Respiratory and hemodynamic parameters

Parameter	Foam mattress			Specialist mattress			ANOVA		
	
	Baseline	PP, 1 h	PP, 6 h	Baseline	PP, 1 h	PP, 6 h	Time	Group	Interaction
PaO_2_/FiO_2_	142 ± 63	178 ± 73	217 ± 69*	143 ± 44	206 ± 127*	243 ± 80*	*P *< 0.001	NS	NS
PaO_2 _(mmHg)	45 ± 9	44 ± 9	44 ± 1 0	45 ± 6	46 ± 10	44 ± 11	NS	NS	NS
Q_VA_/Q_T _(%)	37 ± 8	37 ± 9	35 ± 9*	37 ± 8	36 ± 10	34 ± 10*	*P *< 0.05	NS	NS
Paw (cmH_2_O)	18 ± 3.5	18.2 ± 3	18 ± 3	19 ± 4	19 ± 4	18.4 ± 4	NS	NS	NS
Cst (ml/cmH_2_O)	38.4 ± 10	35.4 ± 8*	37.1 ± 9	38.6 ± 11	35.8 ± 8*	37 ± 8	*P *= 0.007	NS	NS
MPAP (mmHg)	27 ± 4	32 ± 7	30 ± 6	27 ± 4	31 ± 7	32 ± 7	NS	NS	NS
PAOP (mmHg)	12.2 ± 3.0	16.7 ± 4.0*	15.7 ± 4.0*	12.5 ± 5.0	15.8 ± 4.0*	15.9 ± 4.0*	*P *= 0.02	NS	NS
CVP (mmHg)	9.6 ± 4.1	13.7 ± 4	13.1 ± 5	9.4 ± 6	13 ± 5.0	13.5 ± 5.0	*P *= 0.02	NS	NS
CI (l/min per m^2^)	4.3 ± 1.1	4.3 ± 1.2	4 ± 0.9	4.5 ± 0.9	4.1 ± 1.3	4.1 ± 0.9	NS	NS	NS
EVLWI (ml/kg per m^2^)	11.2 ± 6.0	11.1 ± 6.2	10 ± 6.5	11.5 ± 7.3	9.5 ± 6.2	10.6 ± 6.0	NS	NS	NS
ITBV (ml)	1140 ± 475	1100 ± 535	942 ± 330	1165 ± 435	972 ± 205	985 ± 225	NS	NS	NS

Values are expressed as mean ± SD.

ANOVA, analysis of variance; CI, cardiac index; Cst, respiratory static compliance; CVP, central venous pressure; EVLWI, indexed extravascular lung water; FiO2, fraction of inspired oxygen; ITBV, intrathoracic blood volume; MPAP, mean pulmonary arterial pressure; PaCO2, arterial CO2 tension; PaO2, arterial oxygen tension; PAOP, pulmonary artery occlusion pressure; Paw, plateau airway pressure; PP, prone positioning; QVA/QT, venous admixture. *P < 0.05 compared with baseline by Tukey's post-hoc test.

**Table 3:** Hepatic and renal variables

Variable	On inclusion	End of protocol
Bilirubin (μmol/l)	14 ± 9	21 ± 17
ASAT (IU/l)	30 [20–40]	27 [18–52]
ALAT (IU/l)	22 [15–34]	24 [17–49]
Creatinine (μmol/l)	72 [58–100]	75 [58–91]
Prothrombin (%)	63 ± 9	61 ± 8

ASAT, aspartate aminotransferase; ALAT, alanine aminotransferase. For bilirubin and prothrombin, data are expressed as mean ± SD. For transaminases and creatinine, data are expressed as median [interquartile range].

## References

[B1] Pelosi P, Brazzi L, Gattinoni L (2002). Prone position in acute respiratory distress syndrome. Eur Respir J.

[B2] Kiefer P, Nunes S, Kosonen P, Takala J (2000). Effect of positive end-expiratory pressure on splanchnic perfusion in acute lung injury. Intensive Care Med.

[B3] Kiefer P, Morin A, Putzke C, Wiedeck H, Georgieff M, Radermacher P (2001). Influence of prone position on gastric mucosal-arterial PCO2 gradients. Intensive Care Med.

[B4] Hering R, Wrigge H, Vorwerk R, Brensing KA, Schroder S, Zinserling J, Hoeft A, Spiegel TV, Putensen C (2001). The effects of prone positioning on intraabdominal pressure and cardiovascular and renal function in patients with acute lung injury. Anesth Analg.

[B5] Hering R, Vorwerk R, Wrigge H, Zinserling J, Schroder S, von Spiegel T, Hoeft A, Putensen C (2002). Prone positioning, systemic hemodynamics, hepatic indocyanine green kinetics, and gastric intramucosal energy balance in patients with acute lung injury. Intensive Care Med.

[B6] Takata M, Wise R, Robotham J (1990). Effects of abdominal pressure on venous return: abdominal vascular zone conditions. J Appl Physiol.

[B7] Malbrain M, Bakajika D (1999). Abdominal pressure in the critically ill: measurement and clinical relevance. Intensive Care Med.

[B8] Diebel L, Wilson R, Dulchavsky S, Saxe J (1992). Effect of increased intra-abdominal pressure on hepatic arterial, portal venous, and hepatic microcirculatory blood flow. J Trauma.

[B9] Matejovic M, Rokyta R, Radermacher P, Krouzecky A, Sramek V, Novak I (2002). Effect of prone position on hepato-splanchnic hemodynamics in acute lung injury. Intensive Care Med.

[B10] Shelton F, Barnett R, Meyer E (1998). Full-body interface pressure testing as a method for performance evaluation of clinical support surfaces. Appl Ergon.

[B11] Ferrell BA, Osterweil D, Christenson P (1993). A randomized trial of low-air-loss beds for treatment of pressure ulcers. JAMA.

[B12] Chiumello D, Cressoni M, De Grandis E, Landi L, Racagni M, D'Adda A, Gattinoni L (2004). The chest-abdomen support in prone position. Intensive Care Med.

[B13] Bernard G, Artigas A, Brigham K, Carlet J, Falke K, Hudson L, Lamy M, LeGall J, Morris A, Spragg R (1994). Report of the American-European Consensus conference on acute respiratory distress syndrome: definitions, mechanisms, relevant outcomes, and clinical trial coordination. Consensus Committee. J Crit Care.

[B14] Gattinoni L, Mascheroni D, Basilico E, Foti G, Pesenti A, Avalli L (1987). Volume/pressure curve of total respiratory system in paralysed patients: artefacts and correction factors. Intensive Care Med.

[B15] Mihm F, Feeley T, Rosenthal M, Lewis F (1982). Measurement of extravascular lung water in dogs using the thermal-green dye indicator dilution method. Anesthesiology.

[B16] Hoeft A, Vincent J (1995). Transpulmonary indicator dilution: an alternative approach for hemodynamic monitoring. Yearbook of Intensive Care and Emergency Medicine.

[B17] Iberti T, Lieber C, Benjamin E (1989). Determination of intra-abdominal pressure using a transurethral bladder catheter: clinical validation of the technique. Anesthesiology.

[B18] Gattinoni L, Tognoni G, Pesenti A, Taccone P, Mascheroni D, Labarta V, Malacrida R, Di Giulio P, Fumagalli R, Pelosi P (2001). Effect of prone positioning on the survival of patients with acute respiratory failure. N Engl J Med.

[B19] Pelosi P, Tubiolo D, Mascheroni D, Vicardi P, Crotti S, Valenza F, Gattinoni L (1998). Effects of the prone position on respiratory mechanics and gas exchange during acute lung injury. Am J Respir Crit Care Med.

[B20] Doig C, Sutherland L, Sandham J, Fick G, Verhoef M, Meddings J (1998). Increased intestinal permeability is associated with the development of multiple organ dysfunction syndrome in critically ill ICU patients. Am J Respir Crit Care Med.

[B21] Deitch E (2001). Role of the gut lymphatic system in multiple organ failure. Curr Opin Crit Care.

[B22] Montgomery A, Stager M, Carrico C, Hudson L (1985). Causes of mortality in patients with the adult respiratory distress syndrome. Am Rev Respir Dis.

[B23] Slutsky A, Tremblay L (1998). Multiple system organ failure. Is mechanical ventilation a contributing factor?. Am J Respir Crit Care Med.

[B24] Bradley S, Bradley G (1947). The effect of increased intra-abdominal pressure on renal function in man. J Clin Invest.

[B25] Diebel L, Wilson R, Dulchavsky S, Saxe J (1992). Effect of increased intra-abdominal pressure on hepatic arterial, portal venous, and hepatic microcirculatory blood flow. J Trauma.

[B26] Malbrain M, Chiumello D, Pelosi P, Wilmer A, Brienza N, Malcangi V, Bihari D, Innes R, Cohen J, Singer P (2004). Prevalence of intra-abdominal hypertension in critically ill patients: a multicentre epidemiological study. Intensive Care Med.

[B27] McAuley DF, Giles S, Fichter H, Perkins GD, Gao F (2002). What is the optimal duration of ventilation in the prone position in acute lung injury and acute respiratory distress syndrome?. Intensive Care Med.

[B28] Quintel M, Pelosi P, Caironi P, Meinhardt J, Luecke T, Herrmann P, Taccone P, Rylander C, Valenza F, Carlesso E (2004). An increase of abdominal pressure increases pulmonary edema in oleic acid-induced lung injury. Am J Respir Crit Care Med.

[B29] Roch A, Michelet P, Lambert D, Delliaux S, Saby C, Perrin G, Ghez O, Bregeon F, Thomas P, Carpentier J (2004). Accuracy of the double indicator method for measurement of extravascular lung water depends on the type of acute lung injury. Crit Care Med.

[B30] Vollman K, Bander J (1996). Improved oxygenation utilizing a prone positioner in patients with acute respiratory distress syndrome. Intensive Care Med.

[B31] Colmenero-Ruiz M, Pola-Gallego de Guzman D, Jimenez-Quintana MM, Fernandez-Mondejar E (2001). Abdomen release in prone position does not improve oxygenation in an experimental model of acute lung injury. Intensive Care Med.

[B32] Gattinoni L, Pelosi P, Suter PM, Pedoto A, Vercesi P, Lissoni A (1998). Acute respiratory distress syndrome caused by pulmonary and extrapulmonary disease. Different syndromes?. Am J Respir Crit Care Med.

